# Antipsychotic Use and Risk of Breast Cancer in Women With Severe Mental Illness: Replication of a Nationwide Nested Case–Control Database Study

**DOI:** 10.1093/schbul/sbae058

**Published:** 2024-04-30

**Authors:** Marco Solmi, Markku Lähteenvuo, Antti Tanskanen, Olivier Corbeil, Ellenor Mittendorfer-Rutz, Christoph U Correll, Jari Tiihonen, Heidi Taipale

**Affiliations:** Department of Psychiatry, University of Ottawa, Ontario, Canada; SCIENCES LAB, Department of Mental Health, The Ottawa Hospital, Ontario, Canada; Ottawa Hospital Research Institute (OHRI), Clinical Epidemiology Program, University of Ottawa, Ontario, Canada; School of Epidemiology and Public Health, Faculty of Medicine, University of Ottawa, Ontario, Canada; Department of Child and Adolescent Psychiatry, Charité Universitätsmedizin, Berlin, Germany; Department of Forensic Psychiatry, University of Eastern Finland, Niuvanniemi Hospital, Kuopio, Finland; Department of Forensic Psychiatry, University of Eastern Finland, Niuvanniemi Hospital, Kuopio, Finland; Department of Clinical Neuroscience, Karolinska Institutet, Stockholm, Sweden; Center for Psychiatry Research, Stockholm City Council, Stockholm, Sweden; Faculty of Pharmacy, Université Laval, Quebec, Canada; Department of Pharmacy, Quebec Mental Health University Institute, Quebec, Canada; Department of Clinical Neuroscience, Karolinska Institutet, Stockholm, Sweden; Department of Child and Adolescent Psychiatry, Charité Universitätsmedizin, Berlin, Germany; Department of Psychiatry, The Zucker Hillside Hospital, Glen Oaks, NY; Department of Psychiatry and Molecular Medicine, Donald and Barbara School of Medicine at Hofstra/Northwell, Hempstead, NY; Department of Forensic Psychiatry, University of Eastern Finland, Niuvanniemi Hospital, Kuopio, Finland; Department of Clinical Neuroscience, Karolinska Institutet, Stockholm, Sweden; Center for Psychiatry Research, Stockholm City Council, Stockholm, Sweden; Department of Forensic Psychiatry, University of Eastern Finland, Niuvanniemi Hospital, Kuopio, Finland; Department of Clinical Neuroscience, Karolinska Institutet, Stockholm, Sweden; Center for Psychiatry Research, Stockholm City Council, Stockholm, Sweden

**Keywords:** prolactin, hyperprolactinemia, schizoaffective disorder, bipolar disorder, psychotic disorder, psychiatry

## Abstract

**Background and hypothesis:**

Breast cancer is more prevalent in women with severe mental illness than in the general population, and use of prolactin-increasing antipsychotics may be a contributing factor.

**Study design:**

A nested case–control study was conducted using the Swedish nationwide registers (inpatient/outpatient care, sickness absence, disability pension, prescribed drugs, cancers). All women aged 18–85 years with schizophrenia/schizoaffective/other nonaffective psychotic disorder/bipolar disorder and breast cancer (cases) were matched for age, primary psychiatric diagnosis, and disease duration with five women without cancer (controls). The association between cumulative exposure to prolactin-increasing/prolactin-sparing antipsychotics and breast cancer was analyzed using conditional logistic regression, adjusted for comorbidities and co-medications.

**Study results:**

Among 132 061 women, 1642 (1.24%) developed breast cancer between 2010 and 2021, at a mean age of 63.3 ± 11.8 years. Compared with 8173 matched controls, the odds of breast cancer increased in women with prior exposure to prolactin-increasing antipsychotics for 1–4 years (adjusted odds ratio [aOR] = 1.20, 95% confidence interval [CI] = 1.03–1.41), and for ≥ 5 years (aOR = 1.47, 95%CI = 1.26–1.71). There were no increased or decreased odds of breast cancer with exposure to prolactin-sparing antipsychotics of either 1–4 years (aOR = 1.17, 95%CI = 0.98–1.40) or ≥5 years (aOR = 0.99, 95%CI = 0.78–1.26). The results were consistent across all sensitivity analyses (ie, according to different age groups, cancer types, and primary psychiatric diagnosis).

**Conclusions:**

Although causality remains uncertain, exposure to prolactin-elevating antipsychotics for ≥ 1 year was associated with increased odds of breast cancer in women with severe mental illness. When prescribing antipsychotics, a shared decision-making process should consider individual risk factors for breast cancer.

## Introduction

Antipsychotics are critical for symptom remission and recovery in people with severe mental illness, including schizophrenia, other nonaffective psychotic disorders, and bipolar disorder.^1–5^ Given the chronic nature of these disorders, long-term antipsychotic treatment is often necessary, requiring careful consideration of the long-term safety of these medications.^6,7^ While antipsychotic efficacy has been linked to blockade of dopamine D2 receptors in the striatum, blockade of these receptors in other circuits is associated with adverse events such as extrapyramidal symptoms and hyperprolactinemia.^7,8^ Increased prolactin levels induced by antipsychotics are due to blockade of D2 receptors within the tuberoinfundibular dopaminergic pathway, resulting in increased prolactin secretion by lactotroph cells of the anterior pituitary.^9^ The risk of hyperprolactinemia varies with specific antipsychotics; it is greater with first-generation antipsychotics and paliperidone/risperidone, less or absent with quetiapine and clozapine, and partial dopamine agonists, such as aripiprazole, may actually reduce prolactin.^10,11^ Importantly, although antipsychotic-induced hyperprolactinemia usually occurs rapidly after treatment initiation, with potential sexual side effects (eg, decreased libido, amenorrhea, galactorrhea) prompting antipsychotic switching, persistent asymptomatic hyperprolactinemia is also common, and concerns about its long-term effects have been raised.^9^

Long-term use of prolactin-increasing antipsychotics has been associated with infertility,^12^ decreased bone mineral density,^13^ and fragility fractures in people with severe mental illness,^14^ and may also contribute to an increased likelihood of breast cancer in women. Notably, an increased incidence of breast cancer has been reported in schizophrenia compared with the general population (standardized incidence ratio = 1.31, 95% CI = 1.14–1.50).^15^ Women with schizophrenia are also half less likely to undergo mammography screening as women without schizophrenia (odds ratio [OR] = 0.50, 95% CI = 0.38–0.64),^16,17^ and the resulting mortality is almost doubled (relative risk = 1.97, 95% CI = 1.38–2.83).^18^ In addition to known risk factors for breast cancer, such as positive family history, reproductive factors (eg, low parity), use of estrogens (eg, hormone replacement therapy), and lifestyle habits (eg, smoking),^19^ elevated prolactin levels have also been associated with an increased risk of breast cancer in postmenopausal women.^20,21^ Although earlier evidence was inconclusive,^22^ results from recent observational studies suggest an association between prolactin-increasing antipsychotics and breast cancer in women with severe mental illness.^23^

A recent meta-analysis of seven observational studies reported a moderate association between the use of any antipsychotic and the incidence of breast cancer (pooled hazard ratio [HR] = 1.39, 95% CI = 1.11–1.73, and pooled OR = 1.37, 95% CI = 0.90–2.09).^24^ Of note, the majority of the included studies were not conducted specifically among women with severe mental illness,^25–28^ and the comparators were sometimes restricted to women without any mental health disorders,^29,30^ raising concerns about confounding by indication. For example, a Danish population-based case–control study of 60 360 women with breast cancer^26^ and a US database cohort study of 914 such cases^27^ reported an 18%–62% increased risk of breast cancer, respectively, with exposure to prolactin-increasing antipsychotics, but only 2%–9.7% of the women had schizophrenia/bipolar disorder. The largest study to date examining the association between breast cancer and prolactin-increasing antipsychotics specifically among women with schizophrenia was conducted in Finland.^31^ This nationwide nested case–control study, including 1069 women with schizophrenia and breast cancer matched with 5339 controls, found that ≥5 years of exposure to prolactin-increasing antipsychotics (adjusted OR [aOR] = 1.56, 95% CI = 1.27–1.92) was associated with a significantly increased odds of breast cancer, while there was no increased odds for breast cancer with prolactin-sparing antipsychotics at any duration of exposure.

Given the paucity of conclusive data on this association and the limited suitability of randomized controlled trials to investigate rare outcomes resulting from long-term drug exposure, the aim of this study was to replicate the previous Finnish study using Swedish nationwide registries. The hypothesis was that a similar association between breast cancer and exposure to prolactin-increasing, but not to prolactin-sparing antipsychotics, would be found.

## Methods

### Study Design, Cohort, Cases, and Controls

This was a nested case–control study using a design similar to a previous report.^31^ The database included all 132 061 women aged 16 years or older with at least one diagnosis of schizophrenia/schizoaffective disorder (n = 25 280), other nonaffective psychotic disorder (n = 42 052), or bipolar disorder (n = 64 729) in Sweden between 2006 and 2021. Diagnoses were obtained from the National Patient Register and the Microdata for Analyses of Social Insurance (MiDAS) including sickness absence and disability pension (International Classification of Diseases [ICD] codes F20 and F25 [ICD-10] and 295 [ICD-8 and ICD-9] for schizophrenia/schizoaffective disorder; F21–F24, F26–F29 [ICD-10] and 297, 298 [ICD-8 and ICD-9] for other nonaffective psychotic disorders; F30–F31 [ICD-10] and 296 [ICD-8 and ICD-9] for bipolar disorder). Data were extracted from the National Patient Register (hospital stays and specialized outpatient care visits), Prescribed Drug Register (prescription drug purchases), and cancer register. As the Prescribed Drug Register was established in July 2005, it limited the start of the follow-up period in this study, because we wanted to ensure at least 4.5 years of follow-up before diagnosis of breast cancer. This meant that the inclusion period for breast cancer started on January 1, 2010, and ended at the end of the data linkage, namely December 31, 2021. All registers were linked by personal identification number (assigned to each resident in Sweden). Data on ethnicity were not collected.

Cases were defined as women with a first diagnosis of malignant breast cancer (and a previous diagnosis of schizophrenia/schizoaffective disorder, other nonaffective psychotic disorder, or bipolar disorder) between 2010 and 2021. The date of cancer diagnosis was the index date, and the age range at diagnosis was set at 18–85 years. All cases had ≥4.5 years of antipsychotic medication use data prior to their index date. Women were excluded if they had any previous cancer diagnosis (other than nonmelanoma skin cancer), HIV diagnosis, mastectomy, or organ transplant.

Using incidence density sampling,^32^ up to five controls without breast cancer were selected from the study database and matched to each case. Matching criteria were age (±1 year), primary psychiatric diagnosis (ie, schizophrenia/schizoaffective disorder, other nonaffective psychotic disorder, bipolar disorder), and time since first diagnosis (±1 year). Controls had the same exclusion criteria as cases and were assigned the same index date as their matching case. Controls could subsequently become cases (after >1 year).

### Exposure

The main exposure measure was exposure to antipsychotics (Anatomic Therapeutic Chemical [ATC] classification code N05A, excluding lithium [N05AN01]), which were categorized as either prolactin-sparing (ie, clozapine, quetiapine, aripiprazole, brexpiprazole, cariprazine) or prolactin-increasing (all other antipsychotics) on the basis of previous evidence.^10,11,14,31,33,34^ Data recorded in the Prescribed Drug Register include date of dispensing, ATC code, drug name, dose strength, package size, number of packages dispensed, and the amount dispensed in defined daily doses (DDDs) as assigned by the World Health Organization (eg, 1 DDD of oral risperidone is equivalent to a dose of 5 mg, and 1 DDD of oral chlorpromazine is equivalent to a dose of 300 mg). Exposure to prolactin-sparing antipsychotics was restricted to the period when they were used without concomitant prolactin-increasing antipsychotics, in which case it was coded as exposure to prolactin-increasing antipsychotics. Antipsychotic use was categorized according to cumulative duration (<1 year, 1–< 5 years, ≥5 years) and cumulative dose (<500, 500–<1000, 1000–<2000, ≥2000 cumulative sum of DDD). The PRE2DUP method was used to calculate the duration of exposure.^35^ This mathematical model uses purchased drug quantities in DDDs and constructs exposure time periods, taking into account variations such as those caused by drug stockpiling or unrecorded drug use during hospitalization. Each antipsychotic (ie, ATC code) exposure period was modeled separately, and these periods were then processed into prolactin-increasing and prolactin-sparing antipsychotic exposure periods. Thus, for a given prolactin-increasing antipsychotic exposure period, an individual could have used more than one prolactin-increasing antipsychotic concurrently or switched from one drug to another. To reduce the likelihood of reverse causality, antipsychotic exposure was recorded up to 1 year prior to the index date (1-year lag window), as such recent exposure is unlikely to influence cancer occurrence.^36,37^

### Covariates

The following covariates were collected from the hospital discharge and prescription registries and adjusted for in the statistical analyses due to their potential confounding effect^19,38–40^: previous diagnoses of cardiovascular disease, asthma/chronic obstructive pulmonary disease, diabetes, substance use disorder, suicide attempt; number of children; use of medications that may potentially affect breast cancer risk (ie, angiotensin system drugs, beta-blockers, digitalis, dihydropyridine calcium channel blockers, loop diuretics, metformin, spironolactone, statins, acetaminophen/paracetamol, opioids, anticholinergic antiparkinsonian drugs, selective serotonin reuptake inhibitors, tricyclic antidepressants); and use of hormone replacement therapy (systemic preparations with estrogen, progesterone, or progestogen and their combinations; the duration of their cumulative use was categorized as nonuse, <1 year, 1–4 years, and ≥5 years). Covariates were measured with the same 1-year lag window as antipsychotic exposure.

### Statistical Analyses

Matched conditional logistic regression analyses were performed (matching group as strata). Multivariable models were adjusted for the above covariates, and matching factors were controlled for by study design. The main analysis was the association between the cumulative duration of exposure to prolactin-increasing and prolactin-sparing antipsychotics and breast cancer, with exposure of <1 year as the reference. Sensitivity analyses were performed with different exposure modes (cumulative dispensed dose in DDD), and by excluding concomitant aripiprazole use time from the exposure duration to prolactin-increasing antipsychotics. Further sensitivity analyses were also performed using cumulative exposure duration as the main exposure for different breast cancer types (ductal adenocarcinoma, lobular adenocarcinoma) and within different subgroups of primary psychiatric diagnosis (schizophrenia/schizoaffective disorder, other nonaffective psychotic disorder, bipolar disorder) and age at index date (<55 years, 55–69 years, ≥70 years). All analyses were conducted using SAS version 9.4 (SAS Institute Inc).

### Role of the Funding Source

The study’s funders had no role in study design, data collection, data analysis, data interpretation, or writing of the report.

## Results

Of 132 061 women with schizophrenia/schizoaffective disorder/other nonaffective psychotic disorder/bipolar disorder, there were 1642 cases (1.24%) of incident breast cancer between 2010 and 2021 included in the study, matched with 8173 controls. Among the cases, there were 234 (14.3%) women with schizophrenia/schizoaffective disorder, 861 (52.4%) with other nonaffective psychotic disorders, and 547 (33.3%) with bipolar disorder (table 1); these were all analyzed together to increase statistical power. The mean age at breast cancer diagnosis in the cases was 63.2 ± 11.7 years (63.3 ± 11.8 years for controls), with a mean duration of psychiatric illness of 19.1 ± 13.1 years. The most common types of breast cancer were ductal adenocarcinoma (n = 1238, 75.4%) and lobular adenocarcinoma (n = 243, 14.8%). Most characteristics were similar between cases and matched controls, but a higher proportion of the cases had used loop diuretics (standardized mean difference = 0.1). The three most commonly used antipsychotics were similar in both groups (ie, olanzapine, quetiapine, risperidone).

**Table 1.** Characteristics of Cases With Breast Cancer and Controls at Index Date

	Controls^a^	Cases	Standardized Mean Difference
	*N *= 8173	*N* = 1642	
Age categories, years, % (*N*)			
<55	24.4 (1996)	24.1 (396)	0.007
55–69	43.9 (3585)	43.5 (715)	0.006
≥70	31.7 (2592)	32.3 (531)	0.013
Mean time since primary psychiatric diagnosis (SD), years	19.0 (13.0)	19.1 (13.1)	0.006
Primary psychiatric diagnosis, % (*N*)			
Schizophrenia/schizoaffective disorder	14.0 (1141)	14.3 (234)	0.008
Other nonaffective psychotic disorder	52.6 (4298)	52.4 (861)	0.003
Bipolar disorder	33.5 (2734)	33.3 (547)	0.003
Type of breast cancer, % (*N*)			
Ductal adenocarcinoma	NA	75.4 (1238)	NA
Lobular adenocarcinoma	NA	14.8 (243)	NA
Adenocarcinoma NOS	NA	8.7 (142)	NA
Carcinoma NOS	NA	0.6 (10)	NA
Other	NA	0.6 (9)	NA
Number of children, % (*N*)			
0	65.1 (5324)	66.3 (1088)	0.024
1	17.6 (1441)	16.3 (268)	0.035
≥2	17.2 (1408)	17.4 (286)	0.005
Somatic comorbidities, % (*N*)			
Cardiovascular disease	34.2 (2796)	34.5 (567)	0.007
Asthma/COPD	9.0 (733)	10.3 (169)	0.045
Diabetes	11.1 (910)	13.5 (221)	0.071
Psychiatric comorbidities, % (*N*)			
Suicide attempt	11.7 (959)	10.5 (172)	0.040
Substance abuse	15.3 (1252)	15.4 (252)	0.001
Concomitant medication use, % (*N*)			
Hormone replacement therapy (systemic only), duration of use, % (*N*)			
Nonuse	79.8 (6518)	77.5 (1273)	0.054
<1 year	8.9 (727)	10.7 (176)	0.060
1–4 years	7.8 (639)	7.7 (126)	0.005
≥5 years	3.5 (289)	4.1 (67)	0.028
Beta-blockers	28.3 (2316)	28.7 (471)	0.008
Dihydropyridine calcium channel blockers	15.9 (1299)	15.4 (253)	0.013
Angiotensine system drugs	23.8 (1944)	25.5 (419)	0.040
Digitalis	0.9 (76)	1.0 (16)	0.005
Spironolactone	3.9 (319)	4.5 (74)	0.030
Statins	22.7 (1856)	22.4 (368)	0.007
Loop diuretics	16.1 (1319)	20.0 (329)	0.100
Opioids	18.9 (1548)	19.4 (319)	0.012
Paracetamol/acetaminophen	19.4 (1586)	19.7 (324)	0.008
Anticholinergic antiparkinsonian drugs	1.0 (81)	1.2 (19)	0.016
Tricyclic antidepressants	6.9 (561)	6.2 (102)	0.026
SSRIs	22.7 (1858)	23.1 (379)	0.008
Metformin	8.0 (652)	9.4 (155)	0.052
Most common antipsychotics used, % (*N*)^b^			
Olanzapine	13.1 (1068)	13.8 (226)	NA
Risperidone	8.2 (667)	11.0 (181)	NA
Quetiapine	9.5 (774)	9.1 (150)	NA
Aripiprazole	4.9 (404)	6.1 (100)	NA
Zuclopenthixol	4.7 (385)	5.1 (83)	NA
Haloperidol	4.5 (364)	4.3 (71)	NA
Perphenazine	3.2 (261)	3.4 (55)	NA
Clozapine	2.7 (217)	3.2 (52)	NA
Paliperidone	0.9 (71)	0.9 (14)	NA
Register source of diagnosis for schizophrenia/schizoaffective/other nonaffective psychotic disorders/bipolar disorder			
Inpatient care	62.6 (5114)	61.7 (1013)	NA
Specialized outpatient care	26.8 (2188)	27.2 (447)	NA
Sickness absence	4.2 (341)	4.0 (66)	NA
Disability pension	6.5 (530)	7.1 (116)	NA

*Note*: COPD, chronic obstructive pulmonary disease; NA, not applicable; NOS, not otherwise specified; SSRI, selective serotonin reuptake inhibitor.

^a^Five controls were matched to each case for age (±1 year), primary psychiatric diagnosis, and time since first diagnosis (±1 year).

^b^A same woman may have used multiple antipsychotics.

A greater proportion of cases used any antipsychotic for 1–< 5 years (n = 354, 21.6%) and ≥ 5 years (n = 510, 31.1%) than controls (n = 1582, 19.4%, and n = 2101, 25.7%), with resulting aORs for breast cancer of 1.32 (95% CI = 1.13–1.53) for 1–< 5 years antipsychotic exposure and 1.52 (95% CI = 1.31–1.76) for ≥ 5 years antipsychotic exposure, compared with the use of <1 year antipsychotic use (reference) (table 2). More cases (n = 300, 18.3%) had been exposed to prolactin-increasing antipsychotics for 1–<5 years than controls (n = 1384, 16.9%); compared with minimal exposure (< 1 year), the aOR for breast cancer was 1.20 (95% CI = 1.03–1.41). A similar association was found for exposure of ≥5 years to prolactin-increasing antipsychotics; there were 413 (25.2%) cases and 1659 controls (20.3%) with such a duration of exposure, resulting in an aOR of 1.47 (95% CI = 1.26–1.71). No association was found between duration of exposure to prolactin-sparing antipsychotics and breast cancer, neither for exposure of 1–<5 years (aOR = 1.17, 95% CI = 0.98–1.40) nor for ≥5 years (aOR = 0.99, 95% CI = 0.78–1.26).

**Table 2.** Association Between Duration of Use of Any or Prolactin-Increasing or Prolactin-Sparing Antipsychotics and Risk of Breast Cancer With 1-Year Lag Window for Exposure

Cumulative Duration of Use	Controls, % (*N*)	Cases, % (*N*)	Unadjusted OR (95% CI)	Adjusted OR (95% CI)^a^
Any antipsychotic				
<1 year	54.9 (4490)	47.4 (778)	reference	reference
1–<5 years	19.4 (1582)	21.6 (354)	1.32 (1.14–1.54)	1.32 (1.13–1.53)
≥5 years	25.7 (2101)	31.1 (510)	1.55 (1.34–1.79)	1.52 (1.31–1.76)
Prolactin-increasing antipsychotics				
<1 year	62.8 (5130)	56.6 (929)	reference	reference
1–<5 years	16.9 (1384)	18.3 (300)	1.23 (1.06–1.44)	1.20 (1.03–1.41)
≥5 years	20.3 (1659)	25.2 (413)	1.51 (1.30–1.76)	1.47 (1.26–1.71)
Prolactin-sparing antipsychotics				
<1 year	85.3 (6971)	83.5 (1371)	reference	reference
1–<5 years	8.9 (724)	10.7 (175)	1.24 (1.04–1.48)	1.17 (0.98–1.40)
≥5 years	5.9 (478)	5.9 (96)	1.02 (0.81–1.30)	0.99 (0.78–1.26)

*Note*: CI, confidence interval; OR, odds ratio.

^a^Adjusted for: substance abuse, suicide attempt, cardiovascular disease, asthma/chronic obstructive pulmonary disease, diabetes, use of opioids, paracetamol/acetaminophen, digitalis, statins, loop diuretics, beta-blockers, dihydropyridine calcium channel blockers, angiotensine system drugs, anticholinergic antiparkinsonian drugs, tricyclic antidepressants, selective serotonin reuptake inhibitors, metformin, number of children, and duration of systemic hormone replacement therapy use. Prolactin-increasing and prolactin-sparing antipsychotics use analyses adjusted for each other.

Compared with cumulative exposure to <500 DDDs, exposure to 500–999 DDDs (aOR = 1.22, 95% CI = 1.00–1.49), 1000–1999 DDDs (aOR = 1.33, 95% CI = 1.10–1.61), and ≥2000 DDDs (aOR = 1.46, 95% CI = 1.25–1.72) of any antipsychotic were associated with increased odds of breast cancer (table 3). Similarly, cumulative exposure to 1000–1999 DDDs and ≥2000 DDDs of prolactin-increasing antipsychotics was more frequent in cases (n = 134, 8.2% and n = 251, 15.3%) than in controls (n = 559, 6.8% and n = 948, 11.6%), with aORs of 1.30 (95% CI = 1.06–1.60) for exposure to 1000–1999 DDDs and 1.47 (95% CI = 1.24–1.74) for exposure to ≥2000 DDDs, compared to a cumulative exposure of <500 DDDs (reference). No association with breast cancer was found for cumulative antipsychotic exposure of 500–999 DDDs. There was also no association between any of the cumulative exposure categories of prolactin-sparing antipsychotics and breast cancer compared with minimal antipsychotic exposure (<500 DDDs).

**Table 3.** Association Between Cumulative Dispensed Defined Daily Doses of Any or Prolactin-Increasing or prolactin-Sparing Antipsychotics and Risk of Breast Cancer With 1-Year Lag Window for Exposure

Cumulative Dispensed DDDs	Controls, % (*N*)	Cases, % (*N*)	Unadjusted OR (95% CI)	Adjusted OR (95% CI)^a^
Any antipsychotic				
<500	66.8 (5461)	60.5 (994)	reference	reference
500–999	7.9 (647)	8.7 (143)	1.24 (1.02–1.50)	1.22 (1.00–1.49)
1000–1999	8.6 (705)	10.1 (166)	1.35 (1.12–1.63)	1.33 (1.10–1.61)
≥2000	16.6 (1360)	20.7 (339)	1.48 (1.27–1.73)	1.46 (1.25–1.72)
Prolactin-increasing antipsychotics				
<500	74.2 (6066)	69.9 (1147)	reference	reference
500–999	7.3 (600)	6.7 (110)	0.99 (0.80–1.23)	0.97 (0.78–1.20)
1000–1999	6.8 (559)	8.2 (134)	1.32 (1.08–1.63)	1.30 (1.06–1.60)
≥2000	11.6 (948)	15.3 (251)	1.49 (1.26–1.77)	1.47 (1.24–1.74)
Prolactin-sparing antipsychotics				
<500	89.5 (7315)	88.0 (1445)	reference	reference
500–999	2.9 (237)	3.4 (55)	1.18 (0.87–1.59)	1.13 (0.84–1.53)
1000–1999	3.0 (242)	3.8 (62)	1.31 (0.98–1.75)	1.26 (0.94–1.69)
≥2000	4.6 (379)	4.9 (80)	1.08 (0.84–1.40)	1.03 (0.80–1.34)

*Note*: DDD, defined daily dose.

^a^Adjusted for: substance abuse, suicide attempt, cardiovascular disease, asthma/chronic obstructive pulmonary disease, diabetes, use of opioids, paracetamol/acetaminophen, digitalis, statins, loop diuretics, beta-blockers, dihydropyridine calcium channel blockers, angiotensine system drugs, anticholinergic antiparkinsonian drugs, tricyclic antidepressants, selective serotonin reuptake inhibitors, metformin, number of children, and duration of systemic hormone replacement therapy use. Prolactin-increasing and prolactin-sparing antipsychotics use analyses adjusted for each other.

In a sensitivity analysis excluding concomitant aripiprazole use time, duration of exposure to prolactin-increasing antipsychotics of 1–<5 years was associated with an aOR for breast cancer of 1.22 (95% CI = 1.05–1.42), and with an aOR of 1.47 (95% CI = 1.26–1.72) for ≥ 5 years exposure (table 4). The association between prolactin-increasing antipsychotics and breast cancer was further stratified by different cancer types, primary psychiatric diagnosis, and age group at the index date (table 5). Only cumulative exposure duration of ≥5 years to prolactin-increasing antipsychotics was associated with increased odds of both ductal adenocarcinoma (aOR = 1.40, 95% CI = 1.17–1.68) and lobular adenocarcinoma (aOR = 1.65, 95% CI = 1.11–2.45). Among women with schizophrenia/schizoaffective disorder, only exposure to ≥5 years of prolactin-increasing antipsychotics was associated with breast cancer (aOR = 1.48, 95% CI = 1.02–2.13). For other nonaffective psychotic disorders, exposure of 1–<5 years (aOR = 1.42, 95% CI = 1.16–1.74) and ≥5 years (aOR = 1.50, 95% CI = 1.24–1.81) to prolactin-increasing antipsychotics were both associated with increased breast cancer odds. Among women with bipolar disorder, no association was found for any of the exposure durations. Finally, elevated odds of breast cancer were also observed across all age groups at diagnosis for exposure to prolactin-increasing antipsychotics of ≥5 years, but not for exposure of 1–<5 years. The resulting ORs for exposure to prolactin-increasing antipsychotics of ≥5 years were 1.57 (95% CI = 1.05–2.34) for women aged <55 years, 1.39 (95% CI = 1.11–1.74) for women aged 55–69 years, and 1.55 (95% CI = 1.20–2.00) for women aged ≥70 years.

**Table 4.** Association Between Duration of Use of Prolactin-Increasing Antipsychotics Excluding Concomitant Aripiprazole Use Time and Risk of Breast Cancer With 1-year Lag Window for Exposure (Sensitivity Analysis)

Cumulative Duration of Use	Controls, % (*N*)	Cases, % (*N*)	Unadjusted OR (95% CI)	Adjusted OR (95% CI)^a^
Prolactin-increasing antipsychotics excluding concomitant aripiprazole use time				
<1 year	63.2 (5168)	57.2 (939)	reference	reference
1–<5 years	17.1 (1397)	18.6(306)	1.24 (1.07–1.45)	1.22 (1.05–1.42)
≥ 5 years	19.7 (1608)	24.2 (397)	1.48 (1.27–1.73)	1.47 (1.26–1.72)

^a^Adjusted for: substance abuse, suicide attempt, cardiovascular disease, asthma/chronic obstructive pulmonary disease, diabetes, use of opioids, paracetamol/acetaminophen, digitalis, statins, loop diuretics, beta-blockers, dihydropyridine calcium channel blockers, angiotensine system drugs, anticholinergic antiparkinsonian drugs, tricyclic antidepressants, selective serotonin reuptake inhibitors, metformin, number of children, and duration of systemic hormone replacement therapy use. Prolactin-increasing and prolactin-sparing antipsychotics use analyses adjusted for each other.

**Table 5.** Association Between Duration of Prolactin-Increasing Antipsychotic Use and Risk of Breast Cancer Stratified by Type of Cancer, Diagnosis of Psychiatric Disorder and Age at Diagnosis, With 1-year Lag Window for Exposure (Sensitivity Analyses)

Cumulative Duration of Use	Controls, % (*N*)	Cases, % (*N*)	Unadjusted OR (95% CI)	Adjusted OR (95% CI)^a^
Type of cancer				
Ductal adenocarcinoma				
<1 year	63.3 (3906)	58.0 (718)	reference	reference
1–<5 years	16.8 (1038)	17.7 (219)	1.18 (0.98–1.41)	1.14 (0.95–1.37)
≥5 years	19.9 (1226)	24.3 (301)	1.46 (1.23–1.74)	1.40 (1.17–1.68)
Lobular adenocarcinoma				
<1 year	61.3 (740)	53.5 (130)	reference	reference
1–<5 years	16.3 (197)	18.5 (45)	1.36 (0.90–2.04)	1.40 (0.92–2.12)
≥ 5 years	22.4 (271)	28.0 (68)	1.58 (1.08–2.31)	1.65 (1.11–2.45)
Primary psychiatric disorder				
Schizophrenia/schizoaffective disorder				
<1 year	35.4 (404)	28.6 (67)	reference	reference
1–<5 years	23.5 (268)	23.5 (55)	1.19 (0.79–1.81)	1.20 (0.78–1.83)
≥5 years	41.1 (469)	47.9 (112)	1.51 (1.05–2.15)	1.48 (1.02–2.13)
Other nonaffective psychotic disorder				
<1 year	55.4 (2381)	45.8 (394)	reference	reference
1–<5 years	19.3 (829)	22.8 (196)	1.45 (1.19–1.77)	1.42 (1.16–1.74)
≥5 years	25.3 (1088)	31.5 (271)	1.56 (1.30–1.87)	1.50 (1.24–1.81)
Bipolar disorder				
<1 year	85.8 (2345)	85.6 (468)	reference	reference
1–<5 years	10.5 (287)	9.0 (49)	0.86 (0.62–1.18)	0.81 (0.58–1.13)
≥5 years	3.7 (102)	5.5 (30)	1.51 (0.98–2.34)	1.41 (0.90–2.20)
Age categories				
<55 years				
<1 year	71.1 (1420)	66.9 (265)	reference	reference
1–<5 years	16.2 (323)	17.7 (70)	1.24 (0.90–1.70)	1.31 (0.95–1.81)
≥5 years	12.7 (253)	15.4 (61)	1.53 (1.04–2.26)	1.57 (1.05–2.34)
55–69 years				
<1 year	60.6 (2173)	55.0 (393)	reference	reference
1–<5 years	16.8 (602)	17.3 (124)	1.18 (0.93–1.49)	1.10 (0.86–1.41)
≥ 5 years	22.6 (810)	27.7 (198)	1.45 (1.17–1.81)	1.39 (1.11–1.74)
≥70 years				
<1 year	59.3 (1537)	51.0 (271)	reference	reference
1–<5 years	17.7 (459)	20.0 (106)	1.34 (1.02–1.75)	1.31 (0.99–1.74)
≥ 5 years	23.0 (596)	29.0 (154)	1.61 (1.26–2.07)	1.55 (1.20–2.00)

^a^Adjusted for: substance abuse, suicide attempt, cardiovascular disease, asthma/chronic obstructive pulmonary disease, diabetes, use of opioids, paracetamol/acetaminophen, digitalis, statins, loop diuretics, beta-blockers, dihydropyridine calcium channel blockers, angiotensine system drugs, anticholinergic antiparkinsonian drugs, tricyclic antidepressants, selective serotonin reuptake inhibitors, metformin, number of children. and duration of systemic hormone replacement therapy use. Prolactin-increasing and prolactin-sparing antipsychotics use analyses adjusted for each other.

## Discussion

In this large nationwide nested case–control study, there was a 20% increased odds of breast cancer in women with schizophrenia, schizoaffective disorder, other nonaffective psychotic disorders, and bipolar disorder who used prolactin-increasing antipsychotics for 1–4 years, and a 47% increased odds in those with cumulative use of 5 years or more. The odds of both ductal (aOR = 1.40) and lobular adenocarcinoma (aOR = 1.65) were increased after exposure to prolactin-increasing antipsychotics for ≥5 years. These findings are consistent with those of a similar nested case–control study previously conducted in Finland, in which the exposure to prolactin-increasing antipsychotics for ≥5 years was associated with a 56% overall increased odds of breast cancer, as well as increased odds for both ductal (aOR = 1.42) and lobular adenocarcinoma (aOR = 2.36), in women with schizophrenia.^31^ In contrast, long-term exposure to prolactin-sparing antipsychotics (ie, clozapine, quetiapine, third-generation antipsychotics, mainly aripiprazole) was not associated with decreased or increased odds of breast cancer in the Finnish nationwide study,^31^ like in the present Swedish nationwide study.

These results are also consistent with those of a recent study using the US IBM MarketScan Commercial and Medicaid databases, in which the use of prolactin-increasing antipsychotics was associated with a 54%–62% increased risk of breast cancer in women aged 18–65 years over a median follow-up of 4 years,^27^ and of an earlier Danish study, which reported an 18% increased odds of breast cancer with long-term use (ie, cumulative exposure equivalent to 10 000 mg of olanzapine) of prolactin-increasing antipsychotics.^26^ However, in both studies, only a minority of the women included had schizophrenia (0.5%–2.4%) or bipolar disorder (0.6%–7.3%), so the observed associations may not adequately account for the increased risk of breast cancer shared by women with these disorders independent of antipsychotic exposure (ie, confounding by indication). Of note, the Danish study reported an association between the use of prolactin-increasing antipsychotics and estrogen-receptor-positive cancers, but no association for estrogen-receptor-negative cancers.^26^ This finding has not been replicated since, and such information was not available in the cancer registry used for the study reported here.

Although there is some support for an association between duration of exposure and cumulative dose of prolactin-increasing antipsychotics and breast cancer, with limited suggestion of a possible dose-response relationship,^26^ evidence that this association may be mediated by hyperprolactinemia and its amplitude is still lacking. While elevated prolactin levels have been associated with an increased risk of breast cancer, this association has so far only been documented in postmenopausal women and, interestingly in view of the results of the previous Danish study, only for estrogen- and progesterone-receptor-positive cancers.^20,26^ In parallel, it may seem counterintuitive that longer duration of breastfeeding (ie, > 12 months), and thus longer durations of elevated prolactin levels, have been associated with a reduced risk of breast cancer in women.^41^ This association may be due, at least in part, to hyperprolactinemia-induced inhibition of estrogen production, because estrogens increase the risk of breast cancer, and thus may not be indicative of a protective effect of prolactin per se.^42^ Additionally, it has been suggested that prolactin may promote breast tumorigenesis by a mechanism involving an imbalance between prolactin receptor long-form homodimers and prolactin receptor long/intermediate-forms heterodimers.^43^ However, despite being biologically plausible, the evidence remains rather inconclusive,^21,44,45^ and epidemiologic studies investigating the association between prolactin-increasing antipsychotics and breast cancer, including this one, have not been able to examine the association with prolactin levels due to their unavailability in most nationwide registries.

This study has many strengths, including access to large nationwide registers of all Swedish residents over 15 years with extensive data on medication exposure, both in terms of lengthy treatment duration and detailed information (eg, package size, number of packages dispensed, amount dispensed in DDDs, concomitant medications). The nested case–control design allowed controlling for multiple risk factors while accounting for the increased risk of breast cancer in women with schizophrenia, other nonaffective psychotic disorders and bipolar disorder by matching all cases to controls within the same cohort. In addition to matching for age, primary psychiatric diagnosis, and illness duration, the analyses were further adjusted for several factors potentially related to breast cancer risk, including substance abuse, previous suicide attempts, number of children, cardiovascular disease, diabetes, asthma and chronic obstructive pulmonary disease, use of hormone replacement therapy, and many other concomitant medications. Several subgroup and sensitivity analyses were also performed to examine the effects of various exposure measures, as well as age at cancer diagnosis, primary psychiatric diagnosis, and cancer type.

Several limitations need to be considered too. First and importantly, the findings of this study do not allow causality to be assessed, mainly due to the case–control study design, although its realization within a nationwide cohort of the entire Swedish population with schizophrenia (and other psychotic disorders) allows for more generalizable results, and the access to multiple registries prevents some of the information biases found in traditional case–control studies. Second, the analyses could not account for potential unmeasured confounders, such as physical activity level, body mass index, alcohol consumption, smoking status, estrogen-receptor status, family history of breast cancer, and other related genetic factors. On the one hand, while some of these factors may indeed be considered as true confounders, that is, affecting both the exposure and the outcome of interest, and may have led to an overestimation of the main association measured (eg, obesity, a risk factor for breast cancer, may motivate the prescription of a more weight-neutral but prolactin-increasing antipsychotic), other factors may also have led to an underestimation. For example, a positive family history of breast cancer and genes such as BRCA1/2, important risk factors for breast cancer, may motivate the selection of a prolactin-sparing antipsychotic, although there was limited evidence of this potential association available during the study period, reducing the likelihood that it would have had an important effect on the results. On the other hand, prolactin-sparing antipsychotics such as quetiapine or clozapine may cause significant weight gain and lead to obesity, which is a known risk factor for breast cancer and still, their use was not associated with increased odds of breast cancer. Also, the use of clozapine is indicated for the most severe (ie, treatment-resistant) cases of schizophrenia, which in turn is typically associated with poor lifestyle habits and more common risk factors for breast cancer, which could be expected to lead to an overestimation of the association between prolactin-sparing antipsychotics and breast cancer. In addition, it could be hypothesized that some of the association between the use of prolactin-increasing antipsychotics and breast cancer is due to more frequent contact with health care professionals and the health care system as a whole among women receiving an antipsychotic, whether with a pharmacist or a prescriber, resulting in an increased likelihood of being screened for breast cancer compared with women with schizophrenia but not receiving an antipsychotic. While this fact could have contributed to the increased odds of breast cancer found in this study with the use of any antipsychotic, this would also have been true for women using prolactin-sparing antipsychotics, where the odds of breast cancer was not increased. Third, the results obtained cannot be generalized to men because the number of male cases identified was not large enough to allow relevant analyses. Finally, the lack of association found in women with bipolar disorder in this study may be due to limited statistical power, as only 49 (9.0%) of the cases within this subgroup had used prolactin-elevating antipsychotics for 1–4 years and 30 (5.5%) had used prolactin-elevating antipsychotics for ≥5 years.

The results of this study, in addition to previous work, have important clinical implications. Beyond the question of whether or not the association between prolonged exposure to prolactin-increasing antipsychotics in women with schizophrenia and other nonaffective psychotic disorders is causal, women receiving prolactin-increasing antipsychotics appear to have significantly increased odds of breast cancer. Is it possible that we, as clinicians, unconsciously tend to prescribe prolactin-increasing antipsychotics to women at increased risk of breast cancer and prolactin-sparing antipsychotics to women less at risk? If this is the case, and if there is no causality between the use of prolactin-increasing antipsychotics and breast cancer, then there is an urgent need for better screening of risk factors, both those that are modifiable (eg, physical inactivity, obesity, excessive alcohol consumption, smoking, use of hormone replacement therapy) and nonmodifiable risk factors (eg, positive family history, low parity, early menarche, late menopause, genes such as BRCA1/2), with appropriate interventions to mitigate some of the associated risks and routine breast cancer screening during treatment. However, the growing evidence supporting an association between prolactin-increasing antipsychotics and long-term events, such as fragility fractures^14^ and breast cancer,^24,31^ and the plausibility of a biological mechanism between increased breast cancer risk and prolactin elevation,^20,21^ also calls for caution in prescribing prolactin-increasing antipsychotics to women. Importantly, the results of this study suggest that exposure to prolactin-increasing antipsychotics for as little as 1 to 4 years is associated with an increased odds of breast cancer. Although the evidence for whether or not hyperprolactinemia and its magnitude are per se associated with increased breast cancer risk is lacking, clinicians should proactively monitor for hyperprolactinemia and breast cancer risk when prescribing prolactin-raising antipsychotics. Beyond the potential long-term risks, the possibility of sexual side effects and their negative impact on patients’ quality of life^46,47^ and medication adherence^48–50^ must also be considered to promote recovery.

## Data Availability

The project utilized data from the REWHARD consortium, supported by the Swedish Research Council (VR grant number. 2017-00624). These data cannot be made publicly available due to privacy regulations. According to the General Data Protection Regulation, the Swedish law SFS 2018:218, the Swedish Data Protection Act, the Swedish Ethical Review Act, and the Public Access to Information and Secrecy Act, these types of sensitive data can only be made available for specific purposes, including research, that meet the criteria for access to these type of sensitive and confidential data as determined by a legal review. Readers may contact Professor Kristina Alexanderson (kristina.alexanderson@ki.se) regarding the data.
